# Neuropsychological Features of Children and Adolescents With Mitochondrial Disorders: A Descriptive Case Series

**DOI:** 10.3389/fpsyt.2022.864445

**Published:** 2022-04-07

**Authors:** Elise Riquin, Magalie Barth, Thomas Le Nerzé, Natwin Pasquini, Clement Prouteau, Estelle Colin, Patrizia Amati Bonneau, Vincent Procaccio, Patrick Van Bogaert, Philippe Duverger, Dominique Bonneau, Arnaud Roy

**Affiliations:** ^1^Department of Child and Adolescent Psychiatry, University Hospital of Angers, Angers, France; ^2^Univ Angers, Université de Nantes, LPPL, SFR Confluences, Angers, France; ^3^Univ Angers, [CHU Angers], INSERM, CNRS, MITOVASC, SFR ICAT, Angers, France; ^4^Department of Genetics, National Reference Center for Mitochondrial Disorders, University Hospital of Angers, Angers, France; ^5^Department of Pediatric Neurology, University Hospital of Angers, Angers, France; ^6^Reference Center for Learning Disabilities, University Hospital of Nantes, Nantes, France

**Keywords:** mitochondrial disorders, child, adolescent, neuropsychological profile, intelligence, executive function

## Abstract

**Background:**

Mitochondrial disorders (MD) are metabolic diseases related to genetic mutations in mitochondrial DNA and nuclear DNA that cause dysfunction of the mitochondrial respiratory chain. Cognitive impairment and psychiatric symptoms are frequently associated with MD in the adult population. The aim of this study is to describe the neuropsychological profile in children and adolescents with MD.

**Methods:**

We prospectively recruited a sample of 12 children and adolescents between February 2019 and February 2020 in the Reference Center for Mitochondrial Disorders of Angers (France). Participants and their parents completed an anamnestic form describing socio-demographic data and completed the WISC-V (Wechsler Intelligence Scale for Children, 5th edition) and the Parent and Teacher forms of the BRIEF (Behavior Rating Inventory of Executive Function).

**Results:**

In our sample, the mean IQ (Intellectual Quotient) score was 87.3 ± 25.3. The score ranged from 52 to 120. Concerning executive functions, a significant global clinical complaint was found for parents (six times more than normal) and to a lesser extent, for teachers (among 3 to 4 times more). Levels of intelligence and executive functioning were globally linked in our cohort but dissociation remains a possibility.

**Conclusion:**

The results of this study show that MD can be associated to neuropsychological disorders in children and adolescents, especially regarding the intelligence quotient and the executive function. Our study also highlights the need for regular neuropsychological assessments in individuals with MD and developing brains, such as children and adolescents.

## Introduction

Mitochondrial disorders (MD) are the most frequent metabolic diseases related to genetic mutations in mitochondrial DNA and nuclear DNA that cause dysfunction of the mitochondrial respiratory chain (1). In the pediatric population, the prevalence of MD goes from 5 to 15 cases per 100,000 children (1). MD are frequently multisystemic and tend to affect the most energy-dependent organs such as brain, heart, muscles, and kidneys, therefore resulting in heterogeneous phenotypes (2–4).

In the pediatric population, MD can lead to psychiatric disorders, especially anxiety and depression, but also poor quality of life. But it can also lead to behavioral disorders (5, 6). Cognitive impairment is a further common feature in adults and children presenting with MD, such as MELAS (Mitochondrial Encephalopathy, Lactic Acidosis, and Stroke-like episodes) syndrome (7).

In pediatric population, it has a main interest to consider this aspect of MD, as the disease occurs in developing brains. Thereby, it may have a significant impact on developmental trajectories but may also lead to (neuro)-psychological disturbances that can affect quality of life. Children and adolescents with MD may present neuropsychological impairments as those observed in adults. It has to highlight the significance of considering them as important clinical signs for the diagnosis of MD in pediatric practice (5). In the pediatric population, psychiatric symptoms are often depression with behavioral disorders but psychomotor and developmental delays are also described (8). To note, assessments of cognitive function show some variation (9). Also, many children show a decline in cognitive function, as the disease progresses (10). Data are lacking on the effects of MD on executive functions in children, but these should be evaluated in children with MD, linked with the fact that the abnormal presence of intra-cerebral metabolites, like lactate, could disturb the development and the functioning of cerebral networks (9). It is also known that executive function disorders are also linked to depression and anxiety in children and adults (11, 12).

The aim of this study was, therefore, to explore intellectual functioning and executive functioning and to describe them in children with MD.

## Patients and Methods

### Study Design

Our study is a monocentric, cross-sectional descriptive, and analytical exploratory study. It consisted of an evaluation of children and adolescents with MD, and was conducted by a psychiatrist, a neuropsychologist, and a pediatrician. The study protocol was approved by the French Committee for the Protection of Subjects involved in Biomedical Research (n° ID RCB: 2019-A00045-52). Complete information about the study were provided, and all subjects (children and their parents) gave their written consent to participate in the study. All patient data were anonymized.

### Individuals

The methodology and the flow chart of our study is described in Riquin et al. (6). Twelve children and adolescents were prospectively recruited from February 2019 to February 2020. They all received information about the study orally during a consultation with a pediatrician (MB) or by post. The inclusion criteria were that the subject was: (i) a child between 6 and 17 years of age, affected with a form of MD defined by the presence either of an abnormality in the mitochondrial respiratory chain or a mutation known to be involved in mitochondrial disease or; (ii) registered with a social security scheme; and (iii) had consented (both child and the parents) to participate in the study and had signed the consent form.

Exclusion criteria were: (i) the child's refusal to participate in the study; (ii) the refusal of parents that their child participate in the study; (iii) the inability of the child to communicate and answer questions to complete the questionnaires; and (iv) if the child had been previously recruited in an intervention study.

The research protocol was presented orally to the children and their families who were supposed to participate to the study. Those who agreed to participate and who met the inclusion criteria received the consent forms to sign were included in the study by the pediatrician.

A form to determine their socio-demographic and anamnestic data, sex, and age at the time of diagnosis was completed by the parents and the children. The diagnosis of mitochondrial pathology (i.e., the abnormalities of the mitochondrial respiratory chain or the genetic variant responsible for the MD) has been specified by the pediatrician. These data were collected in a file by the pediatrician involved in the inclusions.

Data on the medical history and data on the child's clinical status at the time of consultation were collected. All individuals were diagnosed as having molecular diagnosis or mitochondrial respiratory chain complex defects through biochemical enzymatic assays on muscle tissue samples and met the modified MD criteria proposed by Bernier et al. (13). Lactic acidosis was classified as mild, moderate, or severe according to the increase from normal reference values (≥2-, ≥3-, or ≥4-fold increase, respectively, *N* = 0.5–2.2 mmol/L).

The neuropsychological evaluations were carried out by a neuropsychologist, were adapted to children, and had been widely validated in French. The neuropsychological tests were:

– Intellectual efficiency (IQ): WISC-V (Wechsler Intelligence Scale for Children, 5th edition): the WISC-V is composed of 15 subtests and provides three levels of information (14). The Total Scale is used to calculate the Full-Scale Intellectual Quotient (FSIQ) representing general intellectual ability, while the five main indices reflect the child's level of performance for each of the major cognitive domains (Verbal Comprehension, Fluid Reasoning, Visuospatial Abilities, Working Memory, Processing Speed). The time required to complete the test is ~1–1.5 h. The test is used for children from 6 to 16 years and 11 months old. The FSIQ and all indices were taken into consideration (M = 100; SD = 15). The subtests were also taken into consideration (M = 10; SD = 3).
– Executive function (EF): the BRIEF (Behavior Rating Inventory of Executive Function), Parent and Teacher forms, was used (15). Parents and teachers were asked to rate several aspects of child behavior related to executive functioning in everyday life. The BRIEF is a 86 items inventory, rated on a 3-point Likert scale based on the frequency of the child's behavior: “never” (1 point), “sometimes” (2 points), or “often” (3 points). Seventy-two of the 86 items are distributed on eight clinically and theoretically subscales that measure different aspects of EF and generate two composite indices derived from factorial analyses (16): behavioral regulation index (BRI: shift, inhibition, and emotional control) and metacognitive index (MI: plan/organize, initiation, organization of materials, working memory and monitor). The global executive composite (GEC) index supplies an overall measure of executive functioning. The French normative sample led to the same two factors as in the original American standardization (15–17). Individual subscales and raw index scores are converted into standardized T-scores (mean = 50, standard deviation = 10) depending on age and gender, with higher scores indicating worse functioning. T-scores of 65 or above are defined as being in the clinical range. Two supplementary scales guarantee the questionnaire's validity: (1) the inconsistency score indicates the extent to which responses to items assessing similar aspects are inconsistent with the normative sample and (2) the negativity score reflects the extent to which responses may allow an overly negative perception of the behavior of the child's behavior or dysfunctional framework. The BRIEF-Parent questionnaire was completed during the interview, while the Teacher form was given to the parents during the interview with a letter of motivation addressed to the teacher and a stamped return envelope.
– Statistical analysis: All statistical analyses were conducted by using the Statistical Package for Social Sciences (SPSS) for Windows version V25.0 (IBM Corp., Armonk, NY). Descriptive statistics were produced in the forms of means, standard deviations, percentages, and 95% confidence intervals. Analytical statistics were then calculated using parametric tests: BRIEF and WISC V mean scores were compared to usual norm scores using Student's *t*-test. The significance threshold was set at 5%. The analysis of patients was conducted for the entire clinical group. We then analyzed patients one by one. A patient was considered to have a deficit when s/he presented a T-score ≥ 65 on the corresponding BRIEF clinical scale or composite index.

## Results

### Characteristics of the Sample

Twelve children, 10 boys and two girls, were included in this study (Table 1). Mean age at inclusion was 12.8 years ± 2.3 (9, 15) and mean age at diagnosis was 8.8 years ± 2.6 (4.6; 14.3). The main characteristics of each child are described in a previous article (6).

**Table 1:** Molecular and neuropsychological characteristics of the 12 children from our cohort.

**Individuals**	**1**	**2**	**3**	**4**	**5**	**6**	**7**	**8**	**9**	**10**	**11**	**12**
Sex	M	F	M	M	M	M	F	M	M	M	M	M
Age	10	13	15	13	11	15	10	9	15	15	14	15
Molecular diagnosis	m.8306T>C (MT-TK)	Compound heterozygozity in *NDUFB3* c.410T>C p.(Try22Arg) and c.554G>T p.(Gly70^*^)	m.14849 (MT-CYB)	Complex III abnormal ities without pathogenic variant identified	m.13513 G>A (MT-ND5)	m.3243 A>G (MT-TL1)	m13513 G>A	m.15992 A>T (MT-TP)	m.15992 A>T (MT-TP)	m.3243 A>G (MT-TL1)	m.3243 A>G (MT-TL1)	m.11778G >A (MT-ND4)
Mode of inheritance	Maternal inheritance	Autosomal recessive	Maternal inheritance	Unknown	Maternal inheritance	Maternal inheritance	Maternal inheritance	Maternal inheritance	Maternal inheritance	Maternal inheritance	Maternal inheritance	Maternal inheritance
Plasma lactic acidosis	No	Mild	No	Mild	No	Moderate	Mild	Moderate	Mild	No	No	No
Epilepsy						+				+		
**Neuropsychological features**												
IQ			+		NE	+				NE		
Executive function (parental rating)		+	+			+	+	+		+	+	
Executive function (teacher rating)		+	+							+	+	

*M, Male; F, Female; +, presence of a pathological score;*

* *means that the variant results in the premature termination of the protein*.

*Lactic acidosis was classified according to increase from normal reference values (0.5–2.2 mmol/L), i.e., mild, moderate, and severe: ≥2, ≥3, ≥4-fold the normal respectively; IQ, Intellectual Quotient, a pathological score/deficiency was defined as IQ < 60; NE, Not Evaluated (for two patients who could not be investigated)*.

### Neuropsychological Features

#### Intelligence

The IQ tests (WISC-V) were administered to 10 of the 12 children in our sample.

Two children could not be examined using this test because of a combination of difficulties in verbal communication as well as motor difficulties, preventing them from performing some tests. The mean score in our cohort was 87.3 ± 25.3. The lowest score was 52 and the highest was 120. Two children, 20% of the 10 patients assessed with this scale, had a total IQ score significantly below average and located in the area of impairment (QIT <70) (patients 3 and 6). These results are detailed in Table 2.

**Table 2:** Cognitive profile of children and adolescents who completed the WISC-V (*n* = 10), compared to norms.

**WISC-V Subscales**	**Mean**	**Sd**	**Med**	**Mode**	***t*** **-value**	***p***	**Clinical range (%)**
FSIQ	87.3	25.3	79.0	52.0^+^	t(8) = 5.3	0.001*	2 (20)
PSI	81.2	23.1	84.5	105.0	t(9) = 5.7	≤ 0.001^*^	3 (30)
WMI	92.6	27.2	94.0	115.0	t(9) = 6.8	≤ 0.001^*^	3 (30)
FRI	92.5	19.0	89.5	67.0^+^	t(9) = 7.9	≤ 0.001^*^	1 (10)
VSI	91.6	10.9	86.0	78.0	t(9) = 12.6	≤ 0.001^*^	0 (0)
VCI	92.0	22.1	92.0	50.0	t(9) = 6.9	≤ 0.001^*^	2 (20)
Block design	8.5	2.1	7.5	7.0	t(9) = −60.6	≤ 0.001^*^	0 (0)
Similarities	8.5	3.3	8.5	7.0^+^	t(9) = −41.3	≤ 0.001^*^	1 (10)
Matrix reasoning	9.4	4.5	9.0	5.0	t(9) = −28.8	≤ 0.001^*^	0 (0)
Digit span	8.6	4.6	7.5	7.0	t(9) = −28.3	≤ 0.001^*^	2 (20)
Coding	7.7	4.5	8.0^+^	8.0	t(9) = −30.4	≤ 0.001^*^	3 (30)
Vocabulary	8.0	5.0	8.0	2.0	t(9) = −28.0	≤ 0.001^*^	3 (30)
Figure weights	9.0	3.5	8.0	8.0^+^	t(9) = −39.2	≤ 0.001^*^	1 (10)
Visual puzzles	8.7	2.2	9.0	6.0^+^	t(9) = −61.9	≤ 0.001^*^	0 (0)
Picture span	10.1	3.5	9.0	9.0	t(9) = −37.8	≤ 0.001^*^	0 (0)
Symbol search	7.3	3.0	6.0	6.0	t(9) = −45.6	≤ 0.001^*^	1 (10)

*FSIQ, Full Scale Intellectual Quotient; VSI, Visual Spatial Index; VCI, Verbal Comprehension Index; FRI, Fluid Reasoning Index; WMI, Working Memory Index; PSI, Processing Speed Index; sd, standard deviation. Clinical range (%): number (proportion) of patients in the clinically significant range (IQ score ≤ 70 for composite index, and standard subtest score ≤ 4 for subtests)*.

* *The correlation is significant to the level 0.05 (bilateral)*.

We found a significant decrease in all composite IQ scores and all WISC-V subtests compared to standards. The composite IQ indices were, however, overall in the mid-range (90), or about −0.6 standard deviation below the reference standards. One indice had a slightly lower score (<90): the processing speed index (85.0 ± 19.3), which is about one standard deviation below the standards. The highest composite score was the working memory index (92.6 ± 27.2). Regarding the subtest scores, they were generally between the average and the lower average. The two lowest scores, the Code and Symbols tests (−1 SD), corresponded to the two Processing Speed subtests. Few children had deficit index scores: three children had a deficit score (i.e., ≥ 2DS) for working memory (WMI), and three for processing speed (PSI), two for verbal reasoning (VCI) and fluid reasoning (FRI).

### Executive Function

The results of the BRIEF rating scales are described in Table 3.

**Table 3:** BRIEF parent and teacher results in the whole clinical group compared with normative data.

**Scales**	**BRIEF parents (*n* = 12)**					**BRIEF teachers (*n* = 11)**				
	**M**	**SD**	***t*** **-value (df = 11)**	***p***	**Clinical range (%)**	**M**	**SD**	***t*** **-value (df = 10)**	***p***	**Clinical range (%)**
GEC	65.0	13.0	4.0	0.002^*^	7 (58.3)	63.8	20.7	2.2	0.05^*^	4 (36.4)
BRI	65.8	17.6	3.1	0.01^*^	6 (50)	61.0	21.3	1.7	0.12	4 (36.4)
Inhib	62.9	14.7	3.1	0.01^*^	5 (41.7)	55.1	15.4	1.1	0.30	3 (27.3)
Shift	73.4	19.6	4.1	0.002^*^	6 (50)	64.6	22.1	2.2	0.05^*^	5 (45.5)
Emot C	57.3	18.8	1.3	0.21	4 (33.3)	57.7	17.1	1.5	0.17	2 (18.2)
MI	63.2	11.6	3.9	0.002^*^	5 (41.7)	64.1	20.0	2.3	0.04^*^	4 (36.4)
Initiate	62.9	13.5	3.3	0.007^*^	6 (50)	66.6	16.4	3.4	0.007^*^	5 (45.5)
WM	65.5	14.1	3.8	0.003^*^	6 (50)	66.9	25.9	2.2	0.56	5 (45.5)
Plan/O.	61.5	10.2	3.9	0.002^*^	3 (25)	62.2	19.4	2.1	0.06	5 (45.5)
Org. Ma.	53.6	12.4	1.0	0.34	2 (16.7)	63.7	28.8	1.6	0.15	3 (27.3)
Monitor	61.3	11.8	3.3	0.007^*^	5 (41.7)	56.9	13.9	1.7	0.13	4 (36.4)

*BRIEF, Behavioral Rating Inventory of Executive Function; M, mean; SD, standard deviation; df, degree of freedom; Clinical range (%), number (proportion) of patients in the clinically significant range (T-score ≥ 65); Emot. C., emotional control; BRI, Behavioral Regulation Index; WM, working memory; Plan/O., plan/organize; Org. Ma., organization of materials; MI, Metacognition Index; GEC, global executive composite*.

* *Level of significance set at p = 0.05*.

For the 12 BRIEF parent ratings, one of the inconsistency scales was “inconsistent”, 9 were “acceptable”, and 2 were “questionable”. In the case of the inconsistent BRIEF parent rating, after examining the concerned answers, we believe that the differences could have been linked with particularities of the described situations and then were not excluded, as recommended in the test manual (18). Three protocols showed “elevated” negativity scores, one was “questionable” and the remaining were “acceptable”. The parent ratings which had elevated negativity scores, were not excluded from further analyses as they could reflect elevated levels of executive dysfunction in clinical samples, and maybe also the burden of the caregiver (18). For the 11 teacher ratings (one of the children did not attend school), none of the inconsistency scales was “inconsistent”, most were “acceptable”, and 2 were “questionable”. All of the teacher ratings showed “acceptable” negativity scores on the scale.

Parental complaints were globally significant (GEC) compared with the norms and high, i.e., around 1.5 SD (65.0 ± 13.0). The mean parental scores in the 3 composite indices (BRI, MI, and GEC) and in six of the eight subscales (Inhibition, Shift, Initiate, Working Memory, Plan/Organize, Monitor) were significantly higher than the mean scores expected in the reference population (*p* < 0.01 in all cases). The mean teacher scores in the two composite indices (MI and GEC) and only in two of the eight subscales (Shift, Initiate) were significantly higher than the mean scores expected in the reference population (*p* < 0.05 in all cases). The complaints were also high for the teachers (63.8 ± 20.7) at the limit of significance (*p* = 0.05) and with a greater dispersion. These complaints were found in the metacognitive domain (MI) in both contexts (home and school), but the difficulties appeared more severe at home (out of five, four significant clinical scales at home vs. only one at school). There was also a greater dispersion in school scores for the MI. Behavioral regulation difficulties (BRI) were only significant at home, with two of the three scales involved: inhibition and shift. The shift index was the most impaired.

Moreover, in terms of prevalence, most parents reported a significant overall complaint (58.3%, GEC), and the complaints concerned most of the scales. Thus, half of the parents had a complaint on the shift, initiate, and working memory scales, 30–40% for the inhibition, monitor, and emotional control scales, and <30% for the planning/organization and organization of material scales. More than a third of teachers (36.4%) had a significant overall complaint (GEC), and the complaints concerned most scales. Therefore, 40–50% of teachers had a complaint on the shift, initiate, working memory, planning/organization scales, 30–40% on the monitor scale, and <30% on the inhibition, emotional control, and organization of material scales.

#### Neuropsychological Profiles

The results of each of the twelve children for the different tests and scales are given in Table 4. Considering the intelligence, when the FSIQ was low (from 52 to 79, patients 1, 2, 3, 6, and 11), the BRIEF score seemed to confirm a global score (GEC) that was high for both parents and teachers for three patients (patients 2, 3, and 11). For patient 1, the BRIEF scores were low for Teacher but tend to be clinically significant for Parents, and for the patient 6, the BRIEF scores were high for parents but not evaluated for teachers. When the FSIQ was high (more than 100), the results seemed to show a low global score (GEC) for both parents and teachers (patients 4, 9, and 12). However, for patient 8, who had a good IQ score, we found a global executive complaint for parents, but not for teachers. The serum lactic acidosis level did not seem to be linked with specific neuropsychological impairments in our sample.

**Table 4:** Neuropsychological profiles for each child and adolescent included in the study (n=12).

**Individuals**	**1**	**2**	**3**	**4**	**5**	**6**	**7**	**8**	**9**	**10**	**11**	**12**
Sex	M	F	M	M	M	M	F	M	M	M	M	M
Age	10	13	15	13	11	15	10	9	14	15	14	15
WISC, FSIQ	79	74	**52**	105	NE	**56**	NA	113	120	NE	77	110
PSI	92	83	45	105	NE	53	72	105	111	NE	60	86
WMI	85	88	45	112	NE	65	69	115	115	NE	100	132
FRI	85	79	67	118	NE	67	94	112	115	NE	85	103
VSI	86	81	78	105	NE	84	86	100	108	NE	86	102
VCI	92	70	50	103	NE	62	92	111	116	NE	89	108
BRIEF GEC Parents	64	**65**	**79**	51	55	**65**	**66**	**75**	62	**81**	**80**	37
BRIEF GEC Teachers	48	**95**	**91**	59	58	NA	51	47	40	**67**	**96**	50

*M, Male; F, Female; WISC V, Wechsler Intelligence Scale for Children, 5th edition; FSIQ, Full Score Intelligence Quotient; VSI, Visual Spatial Index; VCI, Verbal Comprehension Index; FRI, Fluid Reasoning Index; WMI, Working Memory Index; PSI, Processing Speed Index; BRIEF, Behavior Rating Inventory of Executive Function; NA, Not Applicable (due to too great a level of heterogeneity in the WISC results); NE, Not Evaluated (due to limited function)*.

*Pathological scores.

## Discussion

Little is known about the neuropsychological function in children with MD (5). To our knowledge, this is the first preliminary clinical study to investigate specific neuropsychological features in this population, including both IQ and executive function.

### Intelligence

The WISC-V was administered to ten children in our cohort. Two children could not be analyzed using this test due to limited capabilities, notably in terms of motor and verbal skills (patients 5 and 10). A third child had been evaluated, but the total score could not be calculated due to too great heterogeneity (patient 7). This finding reflects the clinical heterogeneity of children with mitochondrial disorders and should draw attention to the need to broaden evaluation tools by proposing tools that are better adapted to patients with intellectual disabilities, especially in order to enable an exhaustive evaluation of these groups.

A significant decline was observed in all composite IQ scores and the WISC-V subtests when compared to the standard, but, overall, the IQ scores stayed in the average and below-average zone. However, the IQ scores were distributed in a very dichotomous manner within our population. For patients for whom we have an IQ score, four of them had higher scores (above and well above 100: patients 4, 8, 9, and 12) and five of them had low, or even very low, scores (around 70: patients 1, 2, 3, 6, and 11). Thus, cognitive capabilities in our sample group appear to be either very good or low or very low, with very few average scores. In addition, an IQ score indicating a possible intellectual disability (IQ < 70) was found in two children in our cohort (20%), which corresponds to a higher rate than is found in the general population (14). One indice had an average score below 90: processing speed (81.2 ± 23.1). The distinct drop in the processing speed index may correlate with the clinical reality of children suffering from mitochondrial disorders, which very often present as significant susceptibility to fatigue and psychomotor slowness. The particular weakness of this index can also be explained by executive function (EF) problems, which are used especially in the two subtests that form it (Code and Symbols). These two subtests refer to the index of belonging to the processing speed can mobilize the EF because the processing speed is considered by certain authors as a facet/dimension of the EF (19). That said, these are also tests that by definition also strongly mobilize visuomotor coordination skills. Although it is tricky to compare our patients to populations of children with other genetic disorders, while the charts are different, the intelligence levels in our sample group appear to be higher overall than those found in the literature concerning cohorts with genetic disorders (Niemann-Pick or chronic renal diseases) (20, 21).

Compared to literature focusing on mitochondrial disorders in pediatric populations, to our knowledge, only three studies researched the intelligence of children and adolescents with mitochondrial disorders (8, 22, 23). All of these studies used WISC-III or WISC-IV. One of the studies did not specify the version used (22). The study by Eom et al., which was carried out on 70 children, only studied the IQ of 15 children in this cohort, equaling 20% of the initial cohort, due to “limited function”. For these 15 children, this study found a total IQ of 64.6 ± 21.8, which is a score well-below that of our cohort and corresponds to an average level of intellectual disability. This study did not include a description of the clinical characteristics of these 15 children in accordance with their IQ scores, therefore it is difficult to understand this difference between our results and those of Eom et al. Nevertheless, the authors studied the IQ scores in more detail in the context of diffuse cerebral atrophy and the presence of drug-resistant epilepsy. No significant link was made between the IQ scores and the presence or absence of these two factors. In the study by Morava et al., the authors excluded children with an IQ score below 70 to facilitate methodological pairings of the cohort by considering that intellectual disability could be the result of multiple etiologies and that this led to a too-high number of confounding factors. This exclusion criterion seemed to apply to 50 children out of an initial cohort of 68 children, which corresponds to a 73.5% rate of intellectual disability. As the cohort was not described in this study, we cannot compare it to our population to understand such a high rate of intellectual disability. Shurtleff's study analyzed the IQ of 49 patients with a mitochondrial disorder, ranging from 5.1 to 20.8 years old. Forty patients were tested using the WISC, the Wechsler Adult Intelligence Scale (WAIS) or the Wechsler Preschool and Primary Scale of Intelligence (WPPSI). Nine patients (18.4%) were tested using the Vineland Adaptive Behavior Scale (VABS) (if the patient was clinically judged to be too low functioning to complete one-on-one testing). All of the patients tested using the VABS had drug-resistant epilepsy. The total median score, combining the WISC, WPPI, WAIS, and VABS scores, was 85, which is significantly similar to the score that we found in our cohort. Shurtleff et al. compared the groups with (*n* = 24) and without epilepsy (*n* = 25). The group without epilepsy obtained an average IQ score (FSIQ = 100) that was significantly higher than for the group with epilepsy (FSIQ = 67). In our study, only two children (patients 6 and 10) had epilepsy and only one child had drug-resistant epilepsy (patient 10). Although we could not draw a conclusion from such a small cohort, patients 3 and 6 had the lowest IQ scores in the cohort (FSIQ = 52 and 56) and patient 10 could not be tested using the WISC. It should be noted that it was discovered that one of the children in the cohort (patient 3, FSIQ = 52 IQ) had epilepsy just after the study ended. These results are consistent with the results and conclusions of Shurtleff et al. with regard to the detrimental impact of epilepsy on children with mitochondrial disorders.

With regard to EFs, the use of the BRIEF scale in our sample group made it possible to identify a significant overall clinical complaint with a global score (GEC) that is higher for parents (around six times higher than anticipated) and, to a lesser scale, for teachers (around three to four times higher).

The levels of complaints concerning the cognitive and behavioral factors of EFs in assessments by parents and teachers suggest that these children and adolescents have executive disabilities in their day-to-day life. In reports from parents, the difficulties affected the majority of EF categories for, in order of frequency, flexibility, initiation and working memory, but also control and inhibition, and, to a lesser extent, planning/organizing. The BRIEF questionnaires completed by teachers demonstrated scores that were significantly higher in the metacognition index (under the initiation scale) for patients compared to standard data. Overall, teachers tended to highlight less serious disabilities than parents and there appeared to be interindividual heterogeneity that was more marked at school than at home.

All in all, both parents and teachers seemed to mention significant difficulties concerning EFs in daily life, which leaned toward confirming the hypothesis of executive dysfunction in children with mitochondrial disorders. Taking all this into consideration, it should be remembered that these results are still indirect and exclusively linked to the subjective perspective of adults who interact with the children every day.

The study of the patient profiles shows that the EFs and IQ appear to be linked. However, dissociations are sometimes noted between the two for some patients. In fact, one of the patients (patient 1) with a low cognitive level does not seem to have significant executive difficulty, especially when at home. Alternatively, another patient demonstrating a very high cognitive level (patient 8) was described as having very severe dysexecutive syndrome by their parents. Therefore, it is not possible to think of the two as being systematically linked and also encourages further studies on larger cohorts in order to better understand the interactions and links between EF and IQ. Concerning the long term implications of these findings, Eom and Lee (10) described five phases in the developmental deterioration of children with MD: (1) pre-diagnostic initial decline phase; (2) pre-diagnostic accelerated decline phase; (3) post-diagnostic alleviated phase; (4) post-diagnostic reaccelerated decline phase; and (5) post-diagnostic stagnant phase. The executive dysfunction, at least from the point of view of relatives, has potentially essential specific implications, particularly concerning quality of life, social integration and psychological development, given the close links now well-identified between development executive and these dimensions (24). Future studies need to explore this decline linked with evolution of IQ and/or also EF in these population of children and adolescent.

### Limitations

Our sample was small, but as MD constitutes a very rare class of diseases, and in the light of the state of the literature about neuropsychological profiles of this population, our results are of interest in the field. Due to the fatigue and the psychomotor slowness of these children it should be interesting to consider the General Ability index instead of, or in addition to, the global IQ (14). It is also questionable that executive functions were evaluated with questionnaires and an evaluation based on performance tests is recommended for further studies (25). Nevertheless, executive functions were explored for the first time in this population, which could improve, with these preliminary results, the follow-up of these children and their quality of life.

## Conclusion

Our study highlights the need to consider to the neuropsychological features of children and adolescents with MD, especially intelligence and executive functions. Regular neuropsychological assessments are essential because the disease occurs in pediatric individuals. In addition, MD are chronic and progressive diseases, and a regular reassessment appears necessary. Disorders of executive functions, which are linked to anxiety and depression in children and adults according to the literature (11, 12), have received very little attention in cases of individuals affected with MD (26). Over the neuropsychological assessment, a more comprehensive assessment of the child's functioning could be very interesting in MD. As developing prefrontal circuits are exposed to a developmental anomaly or to acquired brain damage, the early vulnerability of children's EF is now proven in many clinical situations (27).

## Data Availability Statement

The raw data supporting the conclusions of this article will be made available by the authors, without undue reservation.

## Ethics Statement

The studies involving human participants were reviewed and approved by French Committee for the Protection of Subjects involved in Biomedical Research (n° ID RCB: 2019-A00045-52). Written informed consent to participate in this study was provided by the participants' legal guardian/next of kin.

## Author Contributions

All authors: substantial contributions to the conception or design of the work, or the acquisition, analysis or interpretation of data for the work; drafting the work or revising it critically for important intellectual content; providing approval for publication of the content, and agreeing to be accountable for all aspects of the work in ensuring that questions related to the accuracy or integrity of any part of the work are appropriately investigated and resolved.

## Conflict of Interest

The authors declare that the research was conducted in the absence of any commercial or financial relationships that could be construed as a potential conflict of interest.

## Publisher's Note

All claims expressed in this article are solely those of the authors and do not necessarily represent those of their affiliated organizations, or those of the publisher, the editors and the reviewers. Any product that may be evaluated in this article, or claim that may be made by its manufacturer, is not guaranteed or endorsed by the publisher.
